# Manual of Practical Morbid Anatomy

**Published:** 1898-03

**Authors:** 


**Manual of Practical Morbid Anatomy.** By H. D. Rolleston, M.D., and A. A. Kanthack, M.D. Pp. xix., 240. Cambridge: At the University Press. 1894.

It seems rather late in the day to comment upon this useful little book; but as no previous notice of it has appeared in this *Journal*, we are glad of an opportunity to express our appreciation of its value. Not only is the book a most trustworthy guide to the methods of procedure adopted in the *post-mortem* room, but most of the morbid conditions seen in diseased organs are referred to and often briefly described. No intelligent student could use this as a hand-book without acquiring a sound basis of knowledge of morbid anatomy. The book displays at once the wide experience of the writers and their appreciation of those leading features that it is necessary to impress upon the beginner.

